# Pericardial synovial sarcoma in a young adult: case report of a rare malignancy

**DOI:** 10.3332/ecancer.2024.1811

**Published:** 2024-12-05

**Authors:** Javeria Haider, Humera Mahmood, Muhammad Faheem, Shaista Khurshid

**Affiliations:** Department of Oncology, Atomic Energy Cancer Hospital NORI, Islamabad, Pakistan

**Keywords:** pericardium, cardiac, heart, synovial sarcoma, systemic chemotherapy, radiotherapy

## Abstract

Synovial sarcoma is a rare mesenchymal tumour that mainly presents in adolescents and adults younger than 30. It is characterised by a translocation between chromosomes X and 18, which leads to the expression of SS18:SSX fusion proteins. Although it can arise from various soft tissues, the lower limb is the most common site of origin. Pericardial synovial sarcoma is an extremely rare primary malignant tumour of the heart with an unclear prognosis. There are only a few cases reported from Pakistan. Here, we report a case of a 33-year-old male who presented with symptoms of chest pain and shortness of breath. The case was discussed in a multi-disciplinary tumour board and the surgeons deferred surgery as it was associated with high-risk mortality and was also refused by the patient so he was first managed with systemic chemotherapy to which he responded very well and was shifted to maintenance therapy using Pazopanib (small molecule tyrosine kinase inhibitors), to which he initially responded but later symptoms started to worsen and there was an interval increase in the size of the lesion. This report aims to share the diagnosis and management of this patient.

## Introduction

Pericardial synovial sarcoma (PSS) is an exceptionally rare malignancy. Only a few cases have been reported so far. Synovial sarcoma (SS) is an uncommon malignant tumour mainly seen in children and adolescents; its 10-year overall survival rate is about 0%–20% [1]. The precise origin of SS remains unclear, but it is associated with a characteristic chromosomal translocation t(X;18)(p11.2;q11.2) [2]. The clinical outcome of PSS is uncertain but most of them are aggressive in nature. The clinical symptoms of the disease are non-specific, with dyspnea and chest pain being the most common presenting symptom [3]. Occasionally, pericardial effusion or cardiac tamponade may develop [4]. Diagnosing SS in these unusual locations is challenging and requires additional, more advanced diagnostic procedures. Although surgery followed by adjuvant radiation is the cornerstone of treatment for sarcomas, in our case, chemotherapy was preferred due to the tumour’s proximity to critical structures.

## Case report

**Figure 1.**
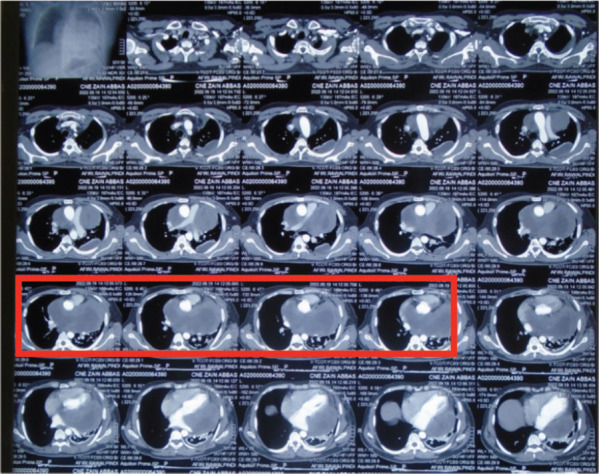
Pre-chemotherapy CECT scan showing a mass in the pericardium that is surrounding the heart and the great vessels and a small pocket of fluid is also seen in the left lung (red box).

**Figure 2.**
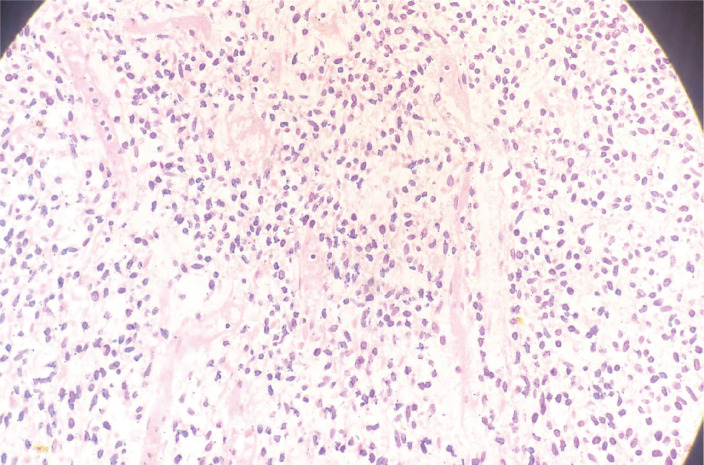
H&E slide of pericardial sarcoma (Histological section examined reveals old spindle-shaped cells arranged in fascicular pattern).

**Figure 3.**
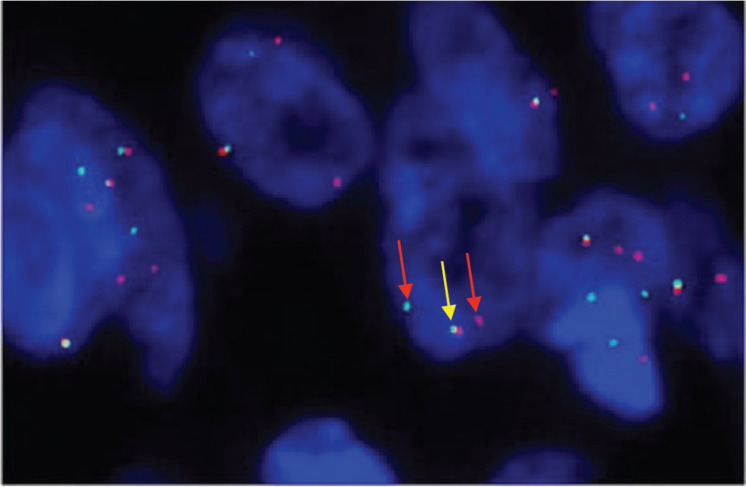
SS 18 FISH showing one fused (yellow colored) and two break-apart (red colored) signals representing gene SS18 gene rearrangement.

A 33-year-old man with a history of 20 pack-years of cigarette smoking and betel nut use, the exact frequency of its use could not be determined but was roughly thrice a day for the past 8 years. He presented with shortness of breath and chest pain at rest which he was experiencing for the past 3 months, and it was progressively increasing in severity. He was first evaluated at a local hospital and was given antibiotics and supportive medicine to which he did not respond. Subsequent evaluation by a cardiologist revealed gross pericardial effusion and a mass obstructing the right atrium on echocardiography. Contrast enhanced computed tomography (CECT) scan confirmed a soft tissue mass within the pericardium that was surrounding the heart and the great vessels measuring roughly 6 × 5.5 × 5 cm and a small pleural effusion in the left lung (Figure 1). At a tertiary care hospital biopsy of the mass was done followed by immunohistochemistry that favoured SS. Furthermore, chromosomal analysis confirmed the diagnosis of SS with t(X;18)(p11.2;q11.2) translocation (Figures 2 and 3). Since the presence of this chromosomal translocation was specific for sarcoma, other differentials were ruled out.

The patient was referred to our oncology department, where a multidisciplinary tumour board (MDT) deemed surgery too risky because of its proximity to great vessels. Instead, chemotherapy with Doxorubicin, Ifosfamide and Mesna was initiated, (with intravenous (IV) doxorubicin calculated at 25 mg/m^2^ IV days 1–3, Ifosfamide 2,500 mg/m^2^ IV days 1–4 and Mesna given first as an IV bolus at 20% of ifosfamide dose followed by continuous infusion of 12–24 hours at 40% of ifosfamide dose, started after completion of ifosfamide infusion) three weekly protocol. The body surface area of our patient was 1.7 kg/m^2^ so the dose of Doxorubicin was 42 mg and ifosfamide was given 3,000 mg (reduced dose). It resulted in significant tumour regression after three cycles. Interim computed tomography (CT) scan showed a marked reduction in the mass and lung effusion (which was not biopsied).

**Figure 4.**
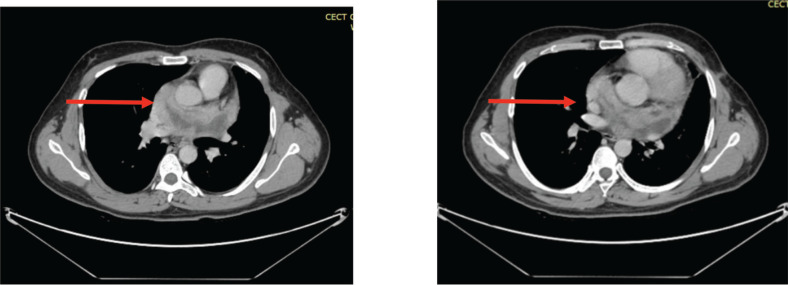
(a&b): CECT scan post six cycles of AIM chemotherapy showing marked interval regression in the size of the pericardial mass (red arrows).

**Figure 5.**
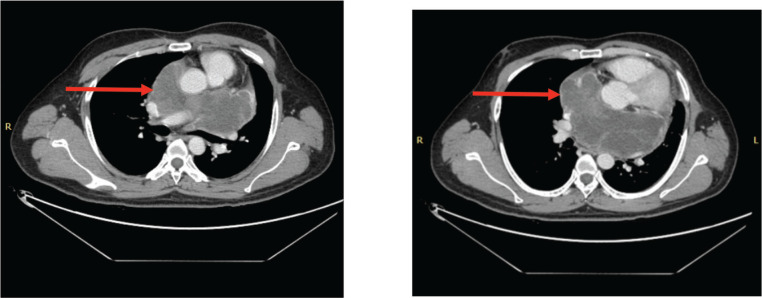
(a&b): CECT scan post six cycles of TKI (Pazopanib) showing an increase in the size of the necrotic component (red arrow).

The patient completed six cycles of chemotherapy (Doxorubicin, Ifosfamide, Mesna) with marked regression in the size of the lesion (Figure 4a,b). He was then shifted to a Tyrosine Kinase Inhibitor, Pazopanib as a cumulative dose of Doxorubicin was achieved which is 450 mg/m^2^. The dose of Pazopanib was 800 mg, given once a day, 1 hour before food intake as consolidation therapy to which he initially responded but later, the patient experienced recurrent chest pain and worsening dyspnea, imaging revealed an increase in the size of necrotic component increasing the overall size of the lesion (Figure 5a,b). The case was discussed multiple times in the MDT for the possibility of surgery but was refused by surgeons on account of proximity to great vessels of the heart. The patient himself was reluctant to go for any surgical procedure. He was also evaluated for the possibility of external beam radiotherapy (EBRT), but the therapeutic dose to the gross tumour could not be prescribed keeping in limit the tolerance doses of organs at risk (OARs) such as heart, aorta and bilateral lungs.

To summarise, our patient diagnosed with PSS who was refused surgery was treated with chemotherapy AIM to which he responded very well and after six cycles, he was shifted to oral TKI (Pazopanib) on which there was no sustained response seen and gradually his symptoms started to reappear. He was then planned on third-line chemotherapy Gemcitabine and Docetaxel which was not started yet.

## Discussion

Cardiac tumours are rare and most of them are accidentally discovered. The incidence ranges from 0.001% to 0.03% [5]. The overwhelming majority of these are benign and only a few are malignant. Malignant cardiac tumours, like PSS, are exceedingly rare, with only a few cases reported in the literature so far; thus, limited data prevent further understanding of this condition [6]. This type of tumour is known to have a poor prognosis with no clarity about the best approach in managing this disease. On average its 10-year overall survival rate is about 0%–20% [1]. The exact origin of SS is unknown but a characteristic chromosomal abnormality t(X;18)( p11.2;q11.2) is seen [6]. PSS, although rare in adults, is known for its aggressive nature and challenging management, often requiring a multidisciplinary approach. Even if multimodality treatment based on surgery, chemotherapy and radiotherapy is performed, the prognosis of PSS remains dismal, with an average survival of 27 months [6].

The most common primary tumour seen in the heart are benign tumours such as myxomas that mostly originate from the right atrium [7]. A few soft-tissue sarcomas such as liposarcoma, rhabdomyosarcoma and angiosarcoma have also been reported but SS of the heart, namely pericardium is extremely rare. PSS generally appears as a solid heterogenous mass with multilocular areas and internal septations [8]. Similarly, in our case, a solid mass showed contrast enhancement on the CT scan was seen. MRI however was not done due to a lack of resources, although it is the best modality for characterising cardiac tumours [8]. Various other mediastinal and pericardial tumours like thymoma and mesothelioma can also resemble PSS thus cytogenetic analysis is necessary for confirmation of diagnosis. Our patient was also labeled as PSS when he tested positive for SS18 gene rearrangement on chromosome 18q11 on the FISH assay [4].

The clinical outcome of PSS is uncertain but most of them are mostly aggressive in nature. The clinical symptoms of the disease are non-specific, with dyspnea and chest pain being the most common presenting symptoms [3]. The patient described here also presented with shortness of breath and chest pain. Gross pericardial effusion was also noted.

Management of SS is challenging and requires a multi-disciplinary approach. Surgical resection is the preferred treatment, but it is often not feasible due to the tumour’s proximity to vital structures [9]. In such cases, alternative options like chemotherapy and (EBRT) are used to downsize the tumour and potentially render it resectable. Table 1 shows a summary of a few cases of PSS reported in the literature highlighting their management plan. However, in our patient, the case was presented multiple times in the MDT, but surgery was not possible due to the tumour’s size and encasement of major vessels. Moreover, the patient himself was not willing to undergo any surgical procedure. Similarly, we were not able to achieve the therapeutic dose to the tumour keeping in limit the tolerance doses of OARs.

Due to the rarity of these tumours, it is very difficult to establish an optimal regimen of chemotherapy. In general, these tumours are generally chemosensitive, particularly to Ifosfamide, with combination regimens like Doxorubicin and Ifosfamide showing higher response rates but with significant toxicity [4, 10]. A case series of 13 patients with SS treated with high-dose Ifosfamide showed a response in all patients, with 4 clinical remissions achieved [10]. Combining doxorubicin with ifosfamide may achieve a higher response rate (58%) but patient tolerability and side effects need to be considered. Similarly, in our patient, there was a marked response seen to Ifosfamide-based chemotherapy.

Pazopanib, a TKI is a well-tolerated drug that achieves a median progression-free survival of 4.6 months as a second-line treatment [12], offers a new treatment avenue for metastatic soft-tissue sarcomas, typically after failure of standard chemotherapy [4, 11]. In this case, Pazopanib was used as maintenance therapy, after a good response to first-line chemotherapy with initial stabilization of the disease, but eventual progression in the necrotic component of the tumour. Among the toxicities of pazopanib, cardiotoxicity must be considered in patients. Since our patient had disease encapsulating the heart, so his electrocardiogram and echocardiography were repeated before each cycle along with hematological and biochemical profiles. Other chemotherapy drugs like Docetaxel, Gemcitabine and Temozolomide can be used as second-line in patients who do not respond to standard or ifosfamide-based treatment but even if they do not result in a sustained response [4, 9].

In our patient after the initial diagnosis, he was given six cycles of chemotherapy using doxorubicin and ifosfamide. Post-chemotherapy there was a regression in tumour size and significant improvement in symptoms. The case was again discussed in a MDT for the possibility of surgery but because of the high risk of morbidity and mortality, it was refused. An external beam radiation plan was also made but it was not possible to give a curative dose to the tumour keeping in limit the tolerance doses of OARs. Due to these restraints, the patient was started on Pazopanib which was reasonably well tolerated but failed to achieve a sustained response and both the size of the disease and symptoms started to worsen.

The majority of the cases reported in the literature were treated using surgery if possible, as seen in Table 1, followed by ifosfamide/doxorubicin-based chemotherapy with no sustained response and very high rates of progression and recurrence as observed in our case where after switching to pazopanib there was no sustained response and the overall size of the lesion again started to increase.

### Clinical outcome

The patient initially received ifosfamide/doxorubicin-based chemotherapy followed by maintenance Pazopanib 800 mg/day and initially the disease remained stable but post 6 months he again developed dyspnea and cough, and CT reported progression in the size of necrotic mass for which he is planned on third line chemotherapy Gemcitabine/Docetaxel.

## Conclusion

The management of SS, particularly in rare locations like the pericardium, is challenging. While surgical resection and EBRT are potential options, chemotherapy plays a critical role when surgery and radiation are not feasible. However, the response and treatment outcomes are better seen in patients who underwent surgical resection which is only possible in a few cases. The prognosis remains poor, with high rates of recurrence and progression, as observed in this case. Ongoing research and case studies are crucial to better understand and manage this rare and aggressive disease.

## Conflicts of interest

The authors declare that there are no competing interests.

## Funding

No funding applicable.

## Ethical approval

Written informed consent was obtained from the patient for publication of this case report and any accompanying images. A copy of the written consent is available for review by the Editor-in-Chief of this journal. It is certified that the case report of a 33-year-old male diagnosed with PSS has been approved by the research and training cell of AECH-NORI.

## Availability of data

The results can be openly accessed.

## Author contributions

JH: Data acquisition, patient selection, writing.

HM: Design, critical decision and final approval.

MF: Analysis, final approval and critical analysis.

SK: histopathological examination of specimen.

## Figures and Tables

**Table 1.** Summarizing the year of publication, treatment and survival of patients with PSS.

Study author	Sex/Age	Year of publication	Treatment summary	Post-treatment status
Duran-Moreno *et al* [6]	56/Male	2019	Incomplete surgical excision f/b six cycles of pegylated doxorubicin 50 mg/m^2^ f/b SBRT^1^ 40 Gy(800 cGy/fr/day) f/b maintenance Pazopanib 800 mg/day).	Patient remained clinically well 36 months after diagnosis.
Boulmay *et al* [1]	19/Female	2007	Surgical resection of tumor.	Patient remained clinically asymptomatic at 4 months post-surgery but did not opt for systemic therapy.
Luo *et al* [2]	19/Male	2022	Surgical excision of mass f/b tumor recurrence after 2 months.	Patient was started on chemotherapy for tumour recurrence and was under treatment.
Bezerra *et al* [3]	29/Female	2013	Emergency pericardial drainage of 100 mL fluid f/b partial surgical excision of mass after 5 months f/b lost to f/u for 9 months.	9 months post-surgery patient was started on chemotherapy but after 2nd cycle she developed pneumonia and died.
Coli *et al* [9]	52/Male	2018	Surgical excision of pericardial tumor with confirmation of malignancy on frozen section f/b four cycles of chemotherapy (ifosfamide/mesna) f/b progression.	Patient was scheduled for artificial heart implant but during this time he developed pulmonary metastases of which he died.
Coli *et al* [9]	39/Male	2018	Surgical excision of pericardial mass f/b histopathological confirmation of disease f/b 6 cycles of chemotherapy (cisplatinum/docexatel) f/b IMRT (54 Gy/25 fractions).	Initially, there was a temporary improvement, but later CT-scan showed extensive growth and patient died 32 months after surgery.
Wu *et al* [10]	45/Female	2013	Patient was initially diagnosed with tuberculous pericarditis and treated with ATT^2^ later imaging revealed a mass in the pericardium which was surgically removed and histologically proven as sarcoma for which she received chemotherapy (doxorubicin/ifosfamide).	Following surgical excision and postoperative adjuvant chemotherapy, the patient has remained clinically free of disease for 32 months.
Phatak *et al* [4]	27/Male	2014	Excisional biopsy f/b neo-adjuvant ifosfamide based chemotherapy f/b surgical excision f/b EBRT.	He had symptom-free survival for 8 months prior to local recurrence. This was managed with left lung upper lobectomy and follow-up chemotherapy with docetaxel. The patient is currently stable with an acceptable functional status.
Chekrine *et al* [11]	13/Male	2014	Partial resection of intrapericardial tumor f/b four cycles ifosfamide/doxorubicin based chemotherapy f/b tumor regression f/b EBRT 30.6 Gy in 17 fractions. Patient remained in remission for 21 months.	The evolution was marked by an anterior superior mediastinal relapse for which the child is receiving second-line chemotherapy combining ifosfamide, carboplatin, and etoposide. After four cycles, the tumor response was less than 50%. The general condition of the patient is under re-evaluation by a team of supportive care.

1 SVRT: Stereotactic body radiotherapy

2 ATT: Anti-tuberculous treatment
